# Imbalance between IL-17A-Producing Cells and Regulatory T Cells during Ischemic Stroke

**DOI:** 10.1155/2014/813045

**Published:** 2014-06-01

**Authors:** Yuehua Hu, Yanhua Zheng, Ya Wu, Bing Ni, Shugui Shi

**Affiliations:** ^1^Department of Neurology, Southwest Hospital, Third Military Medical University, Institute of Interventional Cerebrovascular Disease, Chongqing 400038, China; ^2^Department of Pathology and Experimental Medicine, 306 Hospital of PLA, Beijing 100101, China; ^3^Institute of Immunology PLA, Third Military Medical University, Chongqing 400038, China

## Abstract

Immune responses and inflammation are key elements in the pathogenesis of ischemic stroke (IS). Although the involvement of IL-17A in IS has been demonstrated using animal models, the involvement of IL-17A and IL-17-secreting T cell subsets in IS patients has not been verified, and whether the balance of Treg/IL-17-secreting T cells is altered in IS patients remains unknown. In the present study, we demonstrated that the proportion of peripheral Tregs and the levels of IL-10 and TGF-**β** were reduced in patients with IS compared with controls using flow cytometry (FCM), real-time PCR, and ELISA assays. However, the proportions of Th17 and **γ** **δ** T cells, the primary IL-17A-secreting cells, increased dramatically, and these effects were accompanied by increases in the levels of IL-17A, IL-23, IL-6, and IL-1**β** in IS patients. These studies suggest that the increase in IL-17A-producing cells and decrease in Treg cells might contribute to the pathogenesis of IS. Manipulating the balance between Tregs and IL-17A-producing cells might be helpful for the treatment of IS.

## 1. Introduction

Stroke is the leading cause of death in China and the most frequent cause of permanent disability in adults worldwide. Thromboembolism is the most common mechanism of cerebrovascular occlusion and has been reported to occur as a complication of systemic inflammatory or autoimmune disorders [1]. Inflammation has been increasingly recognized as a key contributor to the pathogenesis of cerebrovascular diseases, particularly ischemic strokes (IS) resulting from arterial occlusion or dynamic insufficiency [2].

Recent evidence suggests that various elements of the immune system are intimately involved in all stages of the ischemic cascade, from acute intravascular events to parenchymal processes that lead to brain damage and tissue repair [3]. Inflammatory mediators, which exaggerate brain edema or directly promote the death of brain cells in the penumbra, can result in the secondary progression of the infarct lesion, which is directly associated with the prognosis of ischemic stroke patients [3]. To prevent the primary and secondary progression of an infarct lesion, an understanding of the detailed mechanism of postischemic inflammation is needed.

T cells are key cells involved in the immune responses [4, 5]. Peripheral T cells are composed of 30~40% CD4^+^ helper T cells, 20~30% *γδ* T cells, and 20~30% CD8^+^T cells [6]. CD4^+^ T cells are classified as Th1, Th2, Th17, and T regulatory (Treg) cells based on their distinct cytokine repertoire [6]. T subsets play important roles in mediating inflammatory responses through the secretion of effector cytokines. IL-17A is one cytokine involved in several inflammation-associated diseases [7], including multiple sclerosis [8], systemic lupus erythematosus [9], and rheumatoid arthritis [10]. Several lymphocyte subsets secrete IL-17A, including Th17 cells and *γδ* T cells [11]. The involvement of IL-17A in IS has been demonstrated in animal models, showing that this cytokine is primarily secreted from *γδ* T cells rather than Th17 cells [12]. However, the involvement of IL-17A and Th17/*γδ* T cells in IS patients remains unclear. In contrast, Treg cells inhibit inflammatory responses [13] and are protective during late stage cerebral ischemia [14]; however, the balance between Tregs and Th17 cells in IS patients is not understood.

Therefore, in the present study, we examined the proportions of IL-17A-producing cells and Tregs and determined the levels of signature transcription factors and cytokines in the peripheral blood of IS patients to characterize the involvement of these cell types in acute IS. These results might provide potential targets for IS therapy and identify potential prognostic indicators.

## 2. Materials and Methods

### 2.1. Patients

T cells are observed in ischemic lesions from day 1, reaching peak levels by day 7 [15]. Therefore, we screened consecutive IS patients between March 2010 and February 2011. Among the 62 patients screened, 12 patients were excluded: 5 patients lived too far from the follow-up exam location, 3 patients met the exclusion criteria, and 4 patients declined informed consent. In addition, we enrolled 30 age-matched individuals as controls. The control subjects had one or more risk factors for cerebral vascular diseases without stroke. Acute IS was defined according to World Health Organization criteria. Patients with subarachnoid hemorrhages, extradural or subdural hemorrhages, transient ischemic attacks, or neurological deficits due to trauma or neoplasms were excluded. Patients with acute infection after stroke were also excluded. Blood samples were collected from IS patients at 7 and 28 days after stroke. In contrast, only one blood sample was obtained from control subjects. The National Institutes of Health Stroke Scale (NIHSS) was used to measure the stroke severity. The NIHSS ranges from 0 to 42, in which a lower score indicates a less severe stroke. Written informed consent was obtained from each patient and control subject prior to study participation, and the study protocol was approved through the Ethics Committee of Southwest Hospital.

### 2.2. Cell Separation and Flow Cytometry

Peripheral blood mononuclear cells (PBMCs) were prepared using a Ficoll density gradient for analysis through flow cytometry (FCM). The cells were labeled with mouse anti-human CD3, CD4, *γδ* TCR, IL-17A, CD25, and FOXP3 (eBioscience, San Diego, CA, USA) monoclonal antibodies or isotype-matched IgG controls and analyzed using FACSort (BD Biosciences Pharmingen, San Jose, CA, USA). For intracellular IL-17A staining, the cells were treated with PMA at 50 ng/mL and ionomycin at 1 *μ*M in the presence of GolgiStop for 4 h. For the FOXP3 analysis, the cells were not stimulated.

### 2.3. ROR*γ*t and FOXP3 Expression Determined through Real-Time PCR

Total RNA was extracted from PBMCs using TRIzol Reagent (Invitrogen, Carlsbad, CA, USA) and quantified through spectrophotometric measurement (NanoDrop; Agilent Technologies, USA). Aliquots of 500 ng were applied as templates for cDNA synthesis using a reverse transcription kit (TaKaRa, Shiga, Japan). GAPDH was amplified as an endogenous control. The PCR reactions were performed in a 25 *μ*L reaction volume containing primers, ROR*γ*t [5′-ACTCAAAGCAGGAGCAATGGAA-3′ (forward) and 5′-AGTGGGAGAAGTCAAAGATGGA-3′ (reverse)] and FOXP3 [5′-AAGGAAAGGAGGATGGACG-3′ (forward) and 5′-CAGGCAAGACAGTGGAAACC-3′ (reverse)], and the SYBR Green kit reagents (TaKaRa). The 2^−ΔΔCT^ method was used to calculate expression relative to the GAPDH housekeeping control [16].

### 2.4. Measurements of Cytokine Levels

Plasma was obtained and stored at −80°C for subsequent analysis of the cytokine levels. The plasma levels of IL-17A, IL-6, IL-10, IL-23, TGF-*β*, and IL-1*β* were determined by use of the Ready-SET-Go ELISA kit (eBioscience, San Diego, CA, USA), following the manufacturer's instructions.

### 2.5. Statistical Analysis

Differences in the numerical values were analyzed using the *t*-test and analysis of covariance (ANCOVA; general linear model univariate analysis of variance), as appropriate. Descriptive statistics for continuous data are expressed as the mean ± SE. A value of *P* < 0.05 was considered statistically significant. All analyses were performed using SPSS13.0 software for Windows.

## 3. Results

### 3.1. Clinical Course of IS Patients and Control Subjects

The demographic characteristics of the 50 patients with ischemic stroke and 30 control patients are summarized in Table 1. There were no significant differences for age or gender, whereas hypertension, diabetes mellitus, and hyperlipidemia were significantly more frequent in stroke compared with the controls.

### 3.2. The IL-17A Protein Levels and Proportion of IL-17A-Producing Cells Were Significantly Elevated in IS Patients

**Figure 1:**
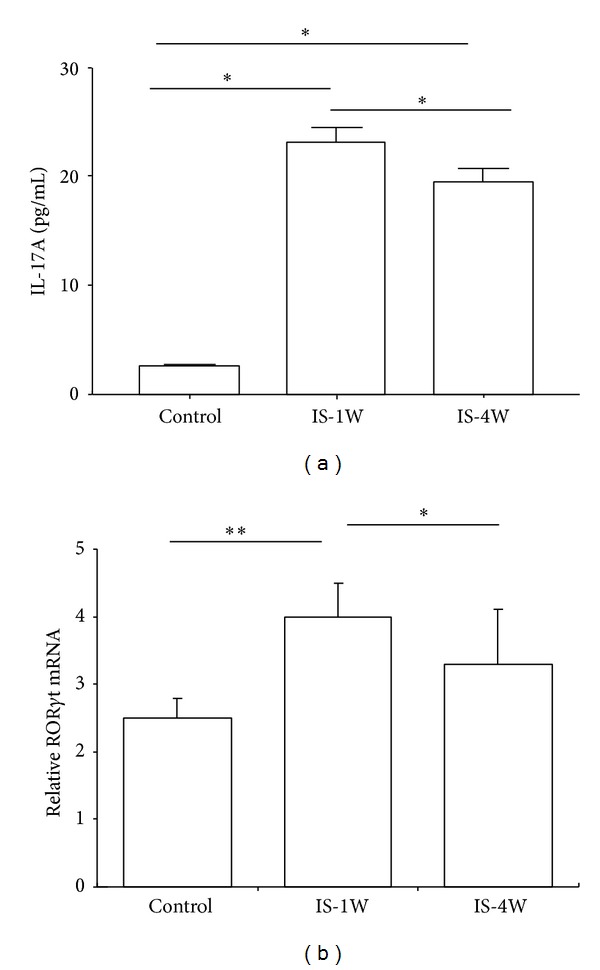
Circulating IL-17A levels and ROR*γ*t mRNA expression were increased in IS patients. (a) Plasma IL-17A protein level. The plasma samples were obtained from IS patients and control subjects at 7 and 28 days after stroke and analyzed using ELISA. (b) ROR*γ*t mRNA levels in PBMCs were obtained from IS patients. The lymphocytes were isolated through the Ficoll-Hypaque separation of the peripheral blood obtained from 30 control subjects and 50 IS patients at 7 and 28 days after stroke. Total RNA was extracted from the PBMCs and the ROR*γ*t mRNA expression levels in the PBMCs obtained from various groups were analyzed using real-time PCR. All mRNA expression values were normalized to GAPDH. *P < 0.05 and **P < 0.01, compared with the controls. IS-1W = 1 week after IS; IS-4W = 4 weeks after IS.

**Figure 2:**
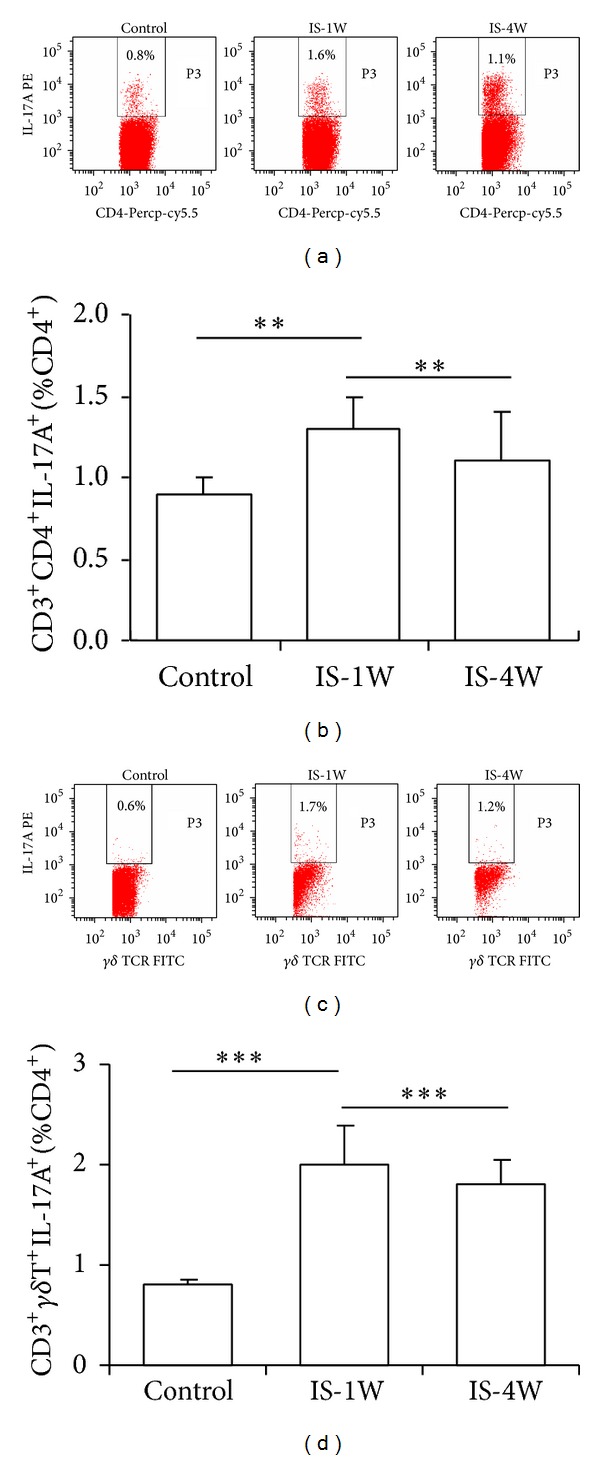
The proportion of IL-17A-producing cells was increased in IS patients. (a) Representative results from the FCM analysis of CD3^+^CD4^+^IL-17A^+^ (Th17) cells among the total CD4^+^ T cell population in IS patients compared with controls. (b) The statistical analysis of the Th17 proportions in 30 control subjects and 50 IS patients at 7 and 28 days after stroke. (c) Representative results from the FCM analysis of inflammatory cells obtained from control subjects and IS patients. The percentage of IL-17A^+^CD3^+^ *γδ* ^+^T cells among the total CD4^+^ T cell population is indicated. (d) Statistical analysis of the results from the FCM analysis of the proportions of IL-17A^+^CD3^+^ *γδ* ^+^ T cells in the blood samples obtained from the 30 control subjects and 50 IS patients at 7 and 28 days after stroke. **P < 0.01 and ***P < 0.001, compared with the controls.

To investigate the potential role for IL-17A in stroke, we analyzed the plasma IL-17A levels in IS patients at 7 and 28 days after stroke and compared with the controls using ELISA (Figure 1). The results showed that IL-17A expression was significantly enhanced in IS patients at 7 days after stroke compared with the controls. Although the IL-17A levels observed at 28 days after stroke were lower than those observed at 7 days, the levels of this cytokine were still higher than those detected in the controls. ROR*γ*t is a key transcription factor for the IL-17A gene; thus, we used real-time PCR to determine whether the expression of ROR*γ*t was altered in accordance with the changes in IL-17A expression. The results showed that the ROR*γ*t mRNA expression levels were significantly increased during IS after 7 days compared with the controls (Figure 1(b)). In contrast, the ROR*γ*t mRNA levels were decreased in IS patients after 28 days, but the mRNA levels were still higher than those observed in control patients (Figure 1(b)). Several lymphocyte subsets secrete IL-17A, including Th17 and *γδ* T cells [11]. Therefore, we determined whether the elevated IL-17A expression reflected altered proportions of these cells during IS. Using FACS analysis, we observed that the proportions of Th17 and *γδ* T cells were markedly increased in the peripheral circulation of patients at 7 days after stroke, but the proportions of these cells were significantly reduced at 28 days after stroke (Figure 2).

### 3.3. The Proportion of Circulating Tregs Was Reduced in IS Patients

**Figure 3:**
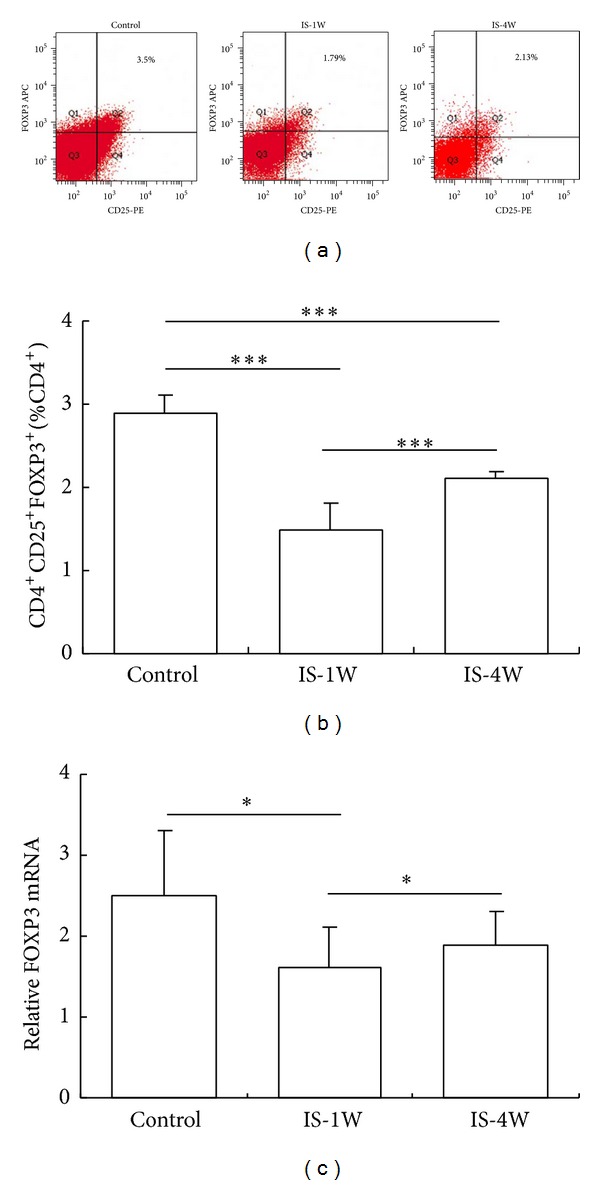
The proportion of CD4^+^CD25^+^FOXP3^+^Tregs was reduced in IS patients. (a) Representative results from the FCM analysis of the samples obtained from control subjects and IS patients. The percentage of CD4^+^CD25^+^FOXP3^+^ T (Treg) cells among the total CD4^+^ T cell population is indicated. (b) Statistical analysis of the results obtained from the FACS analysis of the samples from 30 control subjects and 50 IS patients at 7 and 28 days after stroke. (c) The FOXP3 mRNA expression levels in the PBMCs obtained from various groups were analyzed using real-time PCR, and the values were normalized to GAPDH. *P < 0.05 and ***P < 0.001, compared with the controls. IS-1W = 1 week after IS; IS-4W = 4 weeks after IS.

We used FCM to further examine the proportion of circulating Tregs in IS and control patients. The proportion of Treg cells was significantly reduced in patients at 7 and 28 days after stroke compared with controls. The proportion of Treg cells was higher at 28 days than at 7 days after stroke (Figures 3(a)-3(b)), but this value was still lower than that of the controls. FOXP3 is the primary transcription factor detected in Tregs. FOXP3 mRNA was significantly decreased in patients at 7 and 28 days after stroke compared with controls. Compared with the levels detected after 7 days, the level of FOXP3 mRNA increased significantly after 28 days, but these levels were still lower than those detected in control subjects (Figure 3(c)).

### 3.4. Plasma Cytokine Levels

**Figure 4:**
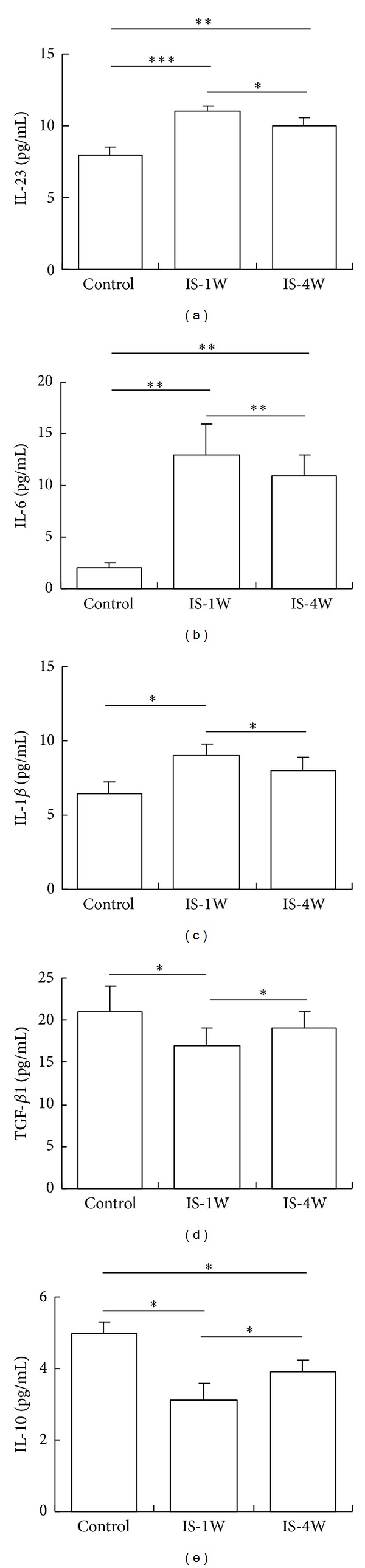
Plasma cytokine levels in patients. The plasma IL-23 (a), IL-6 (b), IL-1*β* (c), TGF-*β* (d), and IL-10 (e) levels in 30 control subjects and 50 IS patients at 7 and 28 days after stroke were analyzed using ELISA. *P < 0.05, **P < 0.01, and ***P < 0.001. IS-1W = 1 week after IS; IS-4W = 4 weeks after IS.

We examined the plasma levels of the signature cytokines produced by Th17 and *γδ*T cells and Treg cells in patients and controls. We observed that the IL-23, IL-6, and IL-1*β* plasma levels were increased in IS patients at 7 and 28 days after stroke compared with controls. Compared with the levels observed after 7 days, the IL-23, IL-6, and IL-1*β* plasma levels were significantly decreased after 28 days, but these levels were still higher than those detected in control subjects (Figures 4(a)–4(c)). The levels of IL-10 and TGF-*β* were markedly decreased in IS patients after 7 days and increased after 28 days; however, these levels were still lower than those detected in control subjects (Figures 4(d)-4(e)).

### 3.5. Risk Factors for T Cell Subset Frequency and Cytokine Levels

We examined potential risk factors affecting IL-17 levels and the frequency of T cell subsets using ANCOVA. The results showed that smoking affects the frequency shift of Th17 and *γδ* ^+^ T cells and IL-17A levels, while hyperlipidemia only affected the frequency shift of *γδ* ^+^ T cells in IS patients (Table 2). Although smoking affected the levels of IL-17A and the frequency of IL-17A-secreting T cells, the percentage of smokers in the IS and control patient groups was comparable (Table 1), suggesting that smoking did not affect the results obtained in the present study. However, the effect of hyperlipidemia on the frequency of *γδ* ^+^ T cells requires further investigation.

## 4. Discussion

Cerebral ischemia leads to the infiltration of major lymphocyte subtypes into the brain [17]. Previous studies have suggested that T cells are important mediators of postischemic inflammation during the delayed phase [18–20]. These infiltrating immune cells produce inflammatory mediators, which exaggerate brain edema or directly promote the death of brain cells in the penumbra, resulting in the secondary progression of the infarct lesion [18]. To prevent the primary and secondary progression of an infarct lesion, a detailed understanding of the postischemic inflammation evoked through these immune cells is needed. Previous studies have shown that there is no difference in activated T cells at 1 day after stroke, but the numbers of these cells peak at 7 days after stroke [15, 21]. In the present study, we examined the changes in the proportions of Tregs, Th17 cells, *γδ* T cells, and related factors in the peripheral blood of stroke patients at 7 and 28 days after stroke as compared with control subjects. We observed an increase in the proportion of IL-17A-producing cells and a decrease in the proportion of Treg cells in IS patients.

Lymphocytes play a crucial role in inflammatory processes, and these cells have been associated with deleterious effects during stroke [4]. However, little is known about the changes in the IL-17A level during the progression of cerebral ischemic disease, particularly in humans. Several studies have indicated that IL-17A levels are elevated in ischemic brain tissue and the peripheral blood of cerebral infarction patients [22, 23]. An experimental stroke model demonstrated that *γδ* T cells are the primary source of IL-17A [12]. The results of the present study revealed that the number of IL-17A-producing cells is greatly elevated in the peripheral blood of patients with IS. Using flow cytometry, we identified *γδ* T and Th17 cells as the primary sources of IL-17A during IS. The results showed that although the proportion of Th17 and *γδ* T cells was lower at 28 days compared with that observed at 7 days after stroke, these cell numbers were higher than those detected in control subjects. Th17 cells secrete IL-17A, and the development of these cells is driven through IL-6 and the transcription factor ROR*γ*t [24]. *γδ* T cells also express ROR*γ*t and secrete IL-17A in response to IL-1*β* and IL-23 [25]. In response to IL-23 induction, IL-17-producing *γδ* T cells act as rapid inflammatory effectors during the delayed phase of brain ischemia [26]. In the present study we observed that IS patients exhibited increased levels of IL-6, IL-23, IL-1*β*, and ROR*γ*t, consistent with the proportions of Th17/*γδ* T cells detected in these patients. IL-23, IL-6, and IL-1*β* are important inflammatory cytokines. IL-6 contributes to tissue repair after ischemic brain injury and IL-23 exerts neurotoxic effects during early-phase IS, while the loss of IL-1*β* function reduces infarct size [27]. In the present study, we observed that IL-23, IL-6, and IL-1*β* plasma levels were increased in IS patients, and these cytokines might promote postischemic inflammation. These results suggest that IL-17A-producing *γδ* T and Th17 cells are inflammatory effectors involved in the delayed phase of stroke patients. Thus, this study is the first report describing the Th17/Treg balance after stroke in human patients.

Tregs secrete the anti-inflammatory cytokines IL-10 and TGF-*β* to suppress the immune response [28], maintain immunologic homeostasis, and prevent autoimmunity [29]. FOXP3 is an important transcription factor for the differentiation of Tregs. However, the results of studies concerning the effects of Tregs on acute ischemic stroke are controversial. Some studies have suggested that Tregs contribute to repair and recovery following experimental stroke [14, 30]. In 2013, Klingenberg et al. demonstrated that Tregs inhibit atherosclerosis. These authors showed that the depletion of Tregs caused a 2.1-fold increase in atherosclerosis without a concomitant increase in vascular inflammation, indicating that FOXP3-expressing Tregs inhibited atherosclerosis through the modulation of lipoprotein metabolism [31]. In contrast, the involvement of regulatory T cells in restricting the area of ischemic brain injury has not been confirmed [32, 33]. In the present study, the proportion of Tregs in the peripheral blood was decreased, and the levels of anti-inflammatory cytokines, namely, IL-10 and TGF-*β*, were lower in IS patients compared with control subjects. These results are consistent with those of previous studies on experimental stroke [34, 35]. Treg cells inhibit inflammatory reactions and inflammatory cytokines inhibit the development and activity of Treg cells [13]. Thus, we hypothesized that Treg cells might exert neuroprotective effects through the suppression of the neurotoxic functions of Th17/*γδ* T cells. Thus, Treg cells might be promising targets for the neuroprotective treatment of IS. The development of strategies targeting T cells might be important to inhibit the inflammatory functions of *γδ* T and Th17 cells or to promote the anti-inflammatory function of Treg cells.

Within a week of experiencing a stroke, all IS patients were initially treated with anti-inflammatory therapy, including antiplatelet drugs, such as aspirin and clopidogrel, and particularly statins [36]. We compared the proportions of Tregs, Th17 cells, and *γδ* T cells and the factors associated with these cell types in patients at 7 and 28 days after stroke. We observed that the proportion of Tregs was increased and the proportion of Th17/*γδ*T cells was decreased on day 7 compared to that on day 28. These results could be attributed to two factors: first, the inflammatory reaction was reduced after 4 weeks, and, second, anti-inflammatory treatment induced cytokine secretion from Tregs and suppressed secretion from Th17/*γδ* T cells, particularly treatment with statins, which suppress proinflammatory reactions [37]. Whether anti-inflammatory therapy impacts the balance between Tregs and Th17/*γδ* T cells needs further study.

In the present study, we investigated the potential roles of Treg/Th17 cells during the onset of stroke. However, the imbalance of Treg/Th17 might occur in patients before stroke. Indeed, previous studies have shown that prolonged hyperlipidemia impairs Treg cell functions and disrupts the balance of Treg/Th17 cells in apoE-Fc*γ*-chain-deficient hyperlipidemic mice [38]. Other studies have also demonstrated that prolonged hypercholesterolemia impaired Tregs in lesions, but the reversal of hypercholesterolemia could prevent the loss of lesional Tregs, suggesting that cholesterol-lowering therapies might induce dynamic and beneficial changes in Treg: effector T cell ratios in atherosclerotic lesions [39]. Taken together, these data indicate that prolonged hyperlipidemia might induce atherosclerosis and consequent stroke through the inhibition of Treg cell function and the induction of the Treg/Th17 cell imbalance.

## 5. Conclusions

In summary, these data suggest an imbalance between IL-17A-producing cells and regulatory T cells in patients with stroke. Thus, regulating the balance between the anti- and proinflammatory effects of T cells might provide a promising therapeutic strategy to improve the recovery from IS.

## Figures and Tables

**Table 1:** Clinical characteristics of the controls and stroke subjects.

	Stroke patients	Controls	*P* value
Number	50	30	
Age	45–76	40–70	NS
Female/male	28/22	15/15	NS
Hypertension	40 (80%)	6 (20%)	*P* < 0.001
Diabetes	32 (64%)	5 (17%)	*P* < 0.001
Smoking	20 (40%)	11 (36%)	NS
Hyperlipidemia	37 (74%)	8 (27%)	*P* < 0.001
Hyperhomocysteinemia	16 (32%)	5 (17%)	NS
NIHSS	2–11	0	
Time since stroke (days)	6.1–7	NA	

NS: not significant; NA: not applicable.

**Table 2:** The risk factors for T cell subset frequency and cytokine levels.

	Hypertension	Diabetes	Smoking	Hyperlipidemia
Th17 cells	NS	NS	*P* < 0.05	NS
*γδ* ^ +^ T cells	NS	NS	*P* < 0.05	*P* < 0.05
Treg cells	NS	NS	NS	NS
IL-17A	NS	NS	*P* < 0.05	NS
IL-6	NS	NS	NS	NS
IL-10	NS	NS	NS	NS
IL-23	NS	NS	NS	NS
TGF-*β*	NS	NS	NS	NS
IL-1*β*	NS	NS	NS	NS

NS: not significant; NA: not applicable.
